# Supplementary material for: The interplay of habitat quality and temperature shape demographic patterns of mule deer (Odocoileus hemionus) in North America
Source: Commun Biol. 2026 Apr 7;9:761. doi: 10.1038/s42003-026-09687-8 (PMC13233836; doi:10.1038/s42003-026-09687-8)
Supplement: Supplementary file 2 — Supplementary Information [file 42003_2026_9687_MOESM2_ESM.pdf]

Supplementary Information

Communications Biology; Article

The interplay of habitat quality and temperature shape demographic patterns of mule deer (*Odocoileus hemionus*) in North America.

William M. Janousek[1*], Aaron N. Johnston[2], Sarah L. Bullock[3], Sarah R. Dewey[4], Embere Hall[5], Teagan A. Hayes[1], Katey S. Huggler[6,7a], Matthew J. Kauffman[8], Tayler N. LaSharr[6], Blake Lowrey[2], Rhiannon P. Jakopak[6], Kevin Monteith[6], Tabitha A. Graves[1]

Affiliations
1. U.S. Geological Survey, Northern Rocky Mountain Science Center, West Glacier, MT, USA.
2. U.S. Geological Survey, Northern Rocky Mountain Science Center, Bozeman, MT, USA.
3. Bureau of Land Management, Renewable Resources Branch, Cheyenne, WY, USA.
4. National Park Service, Grand Teton National Park, Moose, WY, USA.
5. Wyoming Game and Fish Department, 1212 S. Adams Street, Laramie, WY, USA.
6. Haub School of Environment and Natural Resources, Department of Zoology and Physiology, University of Wyoming, Laramie, WY, USA.
7. Wyoming Cooperative Fish and Wildlife Research Unit, Department of Zoology and Physiology, University of Wyoming, Laramie, WY, USA.
8. U.S. Geological Survey, Wyoming Cooperative Fish and Wildlife Research Unit, Department of Zoology and Physiology, University of Wyoming, Laramie, WY, USA.

Current Affiliations

a. Montana Cooperative Wildlife Research Unit, University of Montana, Missoula, MT, USA.

*Corresponding Author: wjanousek@usgs.gov

Supplementary Tables

Supplementary Table 1. Number of animal-years and proportional breakdown of migratory versus non-migratory in resource selection function (RSF) analysis dataset for mule deer in Wyoming, USA from 2000 to 2023.

Study Herd	Animal-years	Percent Migratory	Percent Non-migratory
BatesHole	137	37	63
BatesHoleHatSix	74	17	83
CheyenneRiver	57	3	97
ClarksFork	87	84	16
Douglas	244	57	43
Dubois	110	92	8
MesaRyeGrass	294	94	6
OwlCreekMeeteetse	92	82	18
PlatteValley	68	76	24
PowderPumpkin	286	17	83
RedDesert	882	87	13
SheepMtn	144	52	48
SouthRockSprings	14	0	100
SouthWindRiver	89	66	34
Sweetwater	64	70	30
Tetons	79	100	0
UpperPowderRiver	191	27	73
UpperShoshone	90	92	8
WRIROwlCreek	57	95	5
WRIRWindRiver	137	73	27
WyomingRange	582	92	8

WRIR= Wind River Indian Reservation

Supplementary Table 2. Mean effect and associated credible intervals (CRIs) from seasonal resource selection function (RSF) analyses on mule deer in Wyoming, USA from 2000 to 2023. These data are visualized in Figure 2 of main text.

Season	Variable	Region	Effect	CRI 2.5%	CRI 97.5%
**Summer**	Sagebrush cover	West	-0.09	-0.12	-0.05
		Northeast	0.05	-0.05	0.15
		Southeast	-0.11	-0.18	-0.04
	Non-sage shrub cover	West	0.06	0.03	0.09
		Northeast	0.46	0.35	0.56
		Southeast	0.38	0.32	0.44
	Annual forb & grass cover	West	-0.08	-0.14	-0.01
		Northeast	-0.14	-0.20	-0.09
		Southeast	-0.09	-0.15	-0.03
	Perennial forb & grass cover	West	0.00	-0.03	0.04
		Northeast	0.23	0.15	0.30
		Southeast	0.17	0.11	0.24
	NDVI amplitude	West	0.31	0.26	0.37
		Northeast	0.03	-0.09	0.15
		Southeast	0.13	0.04	0.23
	Rate at peak green-up	West	-0.12	-0.15	-0.08
		Northeast	-0.39	-0.64	-0.13
		Southeast	-0.27	-0.43	-0.12
	Day of peak green-up	West	0.16	0.10	0.21
		Northeast	0.03	-0.14	0.20
		Southeast	0.15	0.03	0.27
	SD curvature	West	0.06	0.02	0.09
		Northeast	0.18	0.09	0.27
		Southeast	0.27	0.21	0.33
	Heat-load index	West	0.06	0.04	0.09
		Northeast	-0.03	-0.08	0.02
		Southeast	0.07	0.02	0.11
	Distance to water	West	-0.48	-0.55	-0.40
		Northeast	-0.16	-0.27	-0.06
		Southeast	-0.31	-0.43	-0.19
	Distance to road	West	-0.18	-0.24	-0.11
		Northeast	-0.09	-0.22	0.03
		Southeast	0.03	-0.07	0.13
**Winter**	Sagebrush cover	Northwest	0.09	0.04	0.14
		Southwest	0.23	0.20	0.25
		Northeast	0.14	0.07	0.21
		Southeast	0.27	0.22	0.33
	Non-sage shrub cover	Northwest	0.08	0.04	0.11
		Southwest	0.09	0.07	0.11

	Northeast	0.09	0.02	0.15
	Southeast	0.28	0.23	0.32
Herbaceous biomass	Northwest	0.03	0.00	0.07
	Southwest	0.00	-0.03	0.04
	Northeast	-0.01	-0.04	0.02
	Southeast	0.04	0.01	0.07
SD curvature	Northwest	0.12	0.08	0.16
	Southwest	0.28	0.25	0.31
	Northeast	0.31	0.26	0.37
	Southeast	0.32	0.27	0.37
Heat-load index	Northwest	0.04	0.01	0.07
	Southwest	0.10	0.08	0.12
	Northeast	-0.01	-0.04	0.02
	Southeast	0.02	-0.01	0.04
Snow-water equivalent	Northwest	-0.12	-0.20	-0.05
	Southwest	-0.02	-0.06	0.01
	Northeast	-0.28	-0.40	-0.15
	Southeast	-0.27	-0.36	-0.18
Distance to road	Northwest	0.05	-0.01	0.11
	Southwest	-0.02	-0.05	0.01
	Northeast	0.02	-0.05	0.08
	Southeast	-0.03	-0.09	0.03

Supplementary Table 3. Mule deer herd units in Wyoming, USA exhibiting significant trends ($p \leq 0.05$) in the amount of premium summer and suitable winter habitat from 2000 to 2023. Trends represent the rate of change per year in the mean proportion of habitat per herd unit.

	Premium Summer Habitat		***Suitable Winter Habitat***	
**Herd Unit**	**Trend**	***P***	**Trend**	***P***
Baggs	0.01	0.05		
Basin	0.06	< 0.01	0.02	< 0.01
Bates Hole/Hat Six	0.03	< 0.01		
Beaver Rim	0.06	< 0.01		
Black Hills	0.04	< 0.01		
Chain Lakes	0.06	0.004		
Cheyenne River	0.05	< 0.01		
Clark's Fork	0.01	< 0.01		
Dubois	0.01	< 0.01		
Ferris	0.02	< 0.01		
Goshen Rim	0.05	0.043		
Greybull River	0.04	< 0.01		
Laramie Mountains	0.03	0.03		
North Bighorn	-0.02	< 0.01		
North Converse	0.08	< 0.01	-0.06	< 0.01
North Natrona	0.07	< 0.01		
Owl Creek/Meeteetse	0.04	< 0.01		
Paintrock	0.02	< 0.01		
Powder River	0.04	< 0.01	0.03	0.04
Project	0.02	< 0.01		
Pumpkin Buttes	0.04	< 0.01		
Rattlesnake	0.06	< 0.01		
Sheep Mountain	0.02	0.04		
Shoshone River	0.01	0.01		
South Rock Springs	0.02	0.03	-0.03	< 0.01
South Wind River	0.02	0.03		
Southwest Bighorn	0.02	< 0.01		
Sweetwater	0.02	< 0.01		
Uinta	0.01	0.02		
Upper Shoshone	0.02	< 0.01	-0.04	0.04
Wyoming Range	0.01	0.03		

Supplementary Table 4. Results from univariate models used for selection of environmental covariates to model age ratios for mule deer in Wyoming, USA from 2000 to 2023. Bolded variables were carried forward to model selection (Supplementary Table 5). CRIs = credible intervals.

	Variable	Effect	CRI 2.5%	CRI 97.5%	looic
**Summer Habitat**	**Proportion premium**	**2.35**	**0.89**	**3.99**	**5934**
	Proportion suitable	0.50	-0.96	1.99	5945
**Winter Habitat**	**Proportion suitable**	**1.31**	**0.01**	**2.67**	**5941**
	Proportion premium	-0.18	-1.50	1.11	5945
**Summer Abiotic**	**Mean summer temperature**	**-2.31**	**-3.85**	**-0.83**	**5929**
**Winter Severity**	**Mean winter temperature**	**2.23**	**1.26**	**3.20**	**5922**
	Accumulated winter severity index	-1.91	-3.02	-0.79	5933
	Snow-water equivalent	-1.00	-2.19	0.22	5944
	Basic winter severity index	-0.12	-1.48	1.23	5946
	Total winter precipitation	-0.38	-1.42	0.69	5946

Supplementary Table 5. Model selection results comparing leave-one-out cross-validation information criterium (looic) across combinations of variables (Supplementary Table 4) used to explain age ratios for mule deer in Wyoming, USA from 2000 to 2023.

Categories	looic
**Sumer temperature + Winter temperature + Summer habitat + Winter habitat**	**5881**
Sumer temperature + Winter temperature	5894
Winter temperature + Summer habitat + Winter habitat	5903
Summer temperature + Summer habitat + Winter habitat	5920
Winter temperature	5922
Summer temperature	5929
Summer habitat + Winter habitat	5930
Summer habitat	5934
Winter habitat	5941

Supplementary Table 6. Results from top model relating environmental covariates to age ratios for mule deer in Wyoming, USA from 2000 to 2023. CRI = credible interval.

Variable	Effect	SE	CRI 2.5%	CRI 97.5%
Intercept	62.57	1.08	60.48	64.75
Summer temperature	-3.39	0.79	-4.98	-1.85
Winter temperature	3.01	0.53	1.97	4.08
Suitable winter habitat	1.22	0.74	-0.22	2.66
Premium summer habitat	2.56	0.92	0.79	4.38

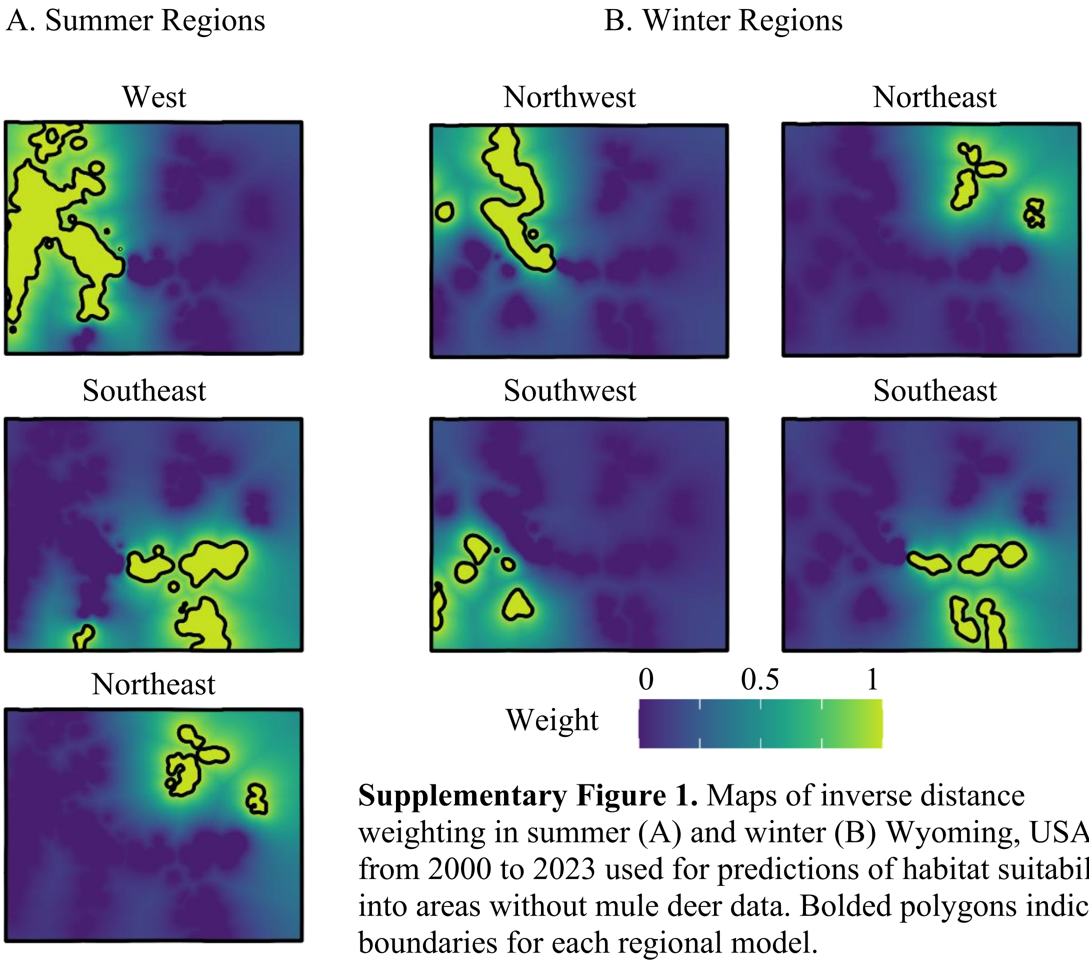

Supplementary Figure 1. Maps of inverse distance weighting in summer (A) and winter (B) Wyoming, USA from 2000 to 2023 used for predictions of habitat suitability into areas without mule deer data. Bolded polygons indicate boundaries for each regional model.

Gauging the effects of summer conditions on age ratios

Here we demonstrate methods used to measure the effects of summer conditions on age ratios of mule deer. Age ratios are estimated on wintering grounds; however, portions of the mule deer populations spend summer on herd units adjacent to their wintering grounds. To account for this seasonal movement and the range of conditions experienced by surveyed deer populations we determined the proportion of deer that summer in adjacent herd units and those that stay within their wintering herd unit using GPS data. We then calculated a weighted average of the summer conditions experienced by mule deer based on those proportions. In the example below we highlight the Upper Shoshone herd unit (USHU). Deer wintering in USHU spend summer in three additional herd units. Of the deer that winter on USHU from our dataset, 83% (n = 69), spend summer in herd units with higher proportions of suitable habitat. Failing to account for these seasonal movements, i.e. assuming all deer wintering in USHU remained there for summer, would underestimate the proportion of suitable habitat experienced by deer during summer. This example is based on habitat suitability in 2020 (Supplementary Figure 2).

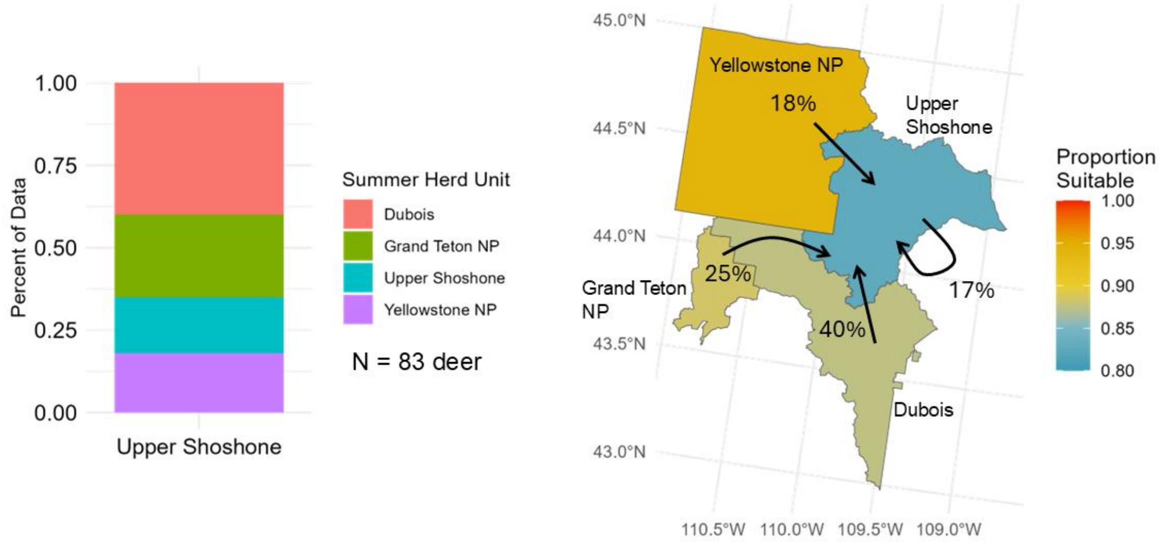

Supplementary Figure 2. Visualizing the methods to account for the variable summer conditions experienced by mule deer in Wyoming, USA that spend summer in different herd units than their wintering grounds.

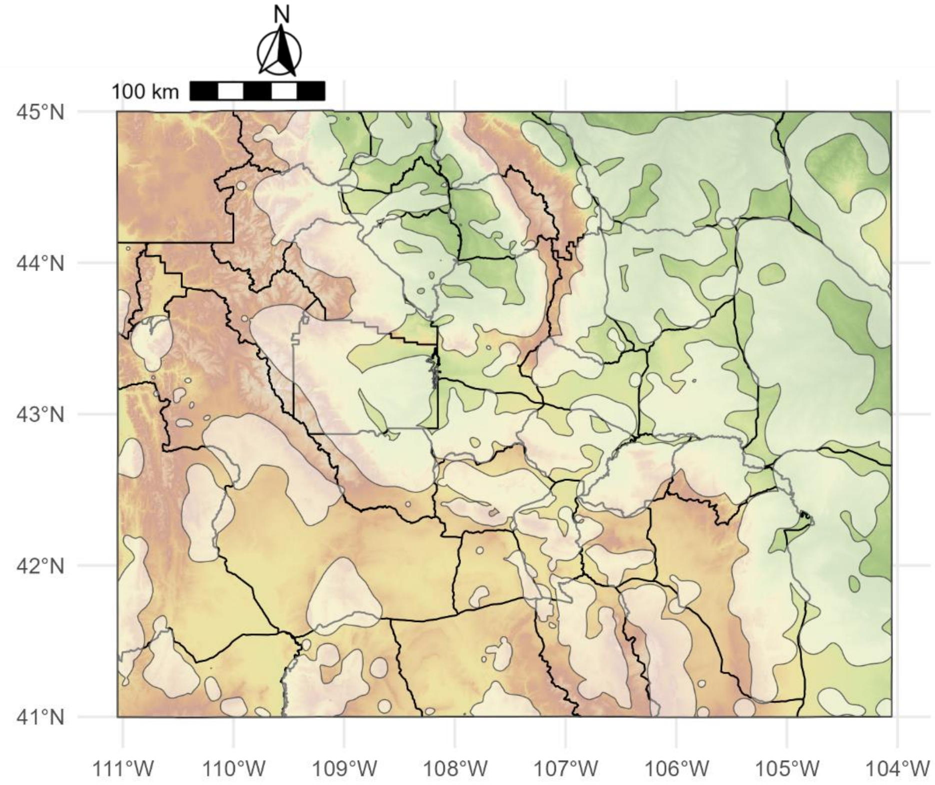

Supplementary Figure 3. Map highlights the areas categorized as winter use areas for mule deer in Wyoming, USA from 2000 to 2023. White shaded polygons are the winter use areas mapped over herd units (black lines) defined by Wyoming Game and Fish. The background relief is based on the SRTM 90m digital elevation model [1].

NIMBLE model code for estimating Resource Selection Functions (RSFs)

This example model is formulated for the winter RSF analysis. To run model for summer covariates, refer to main text and adjust variables as needed. We ran this model separately for each season-region combination. Please see data release for data used in this model [2]. Note that these models may take days to weeks to run depending on processing power.

```
# Load the data
df <- "path to data file"

NimModel <- nimbleCode({
  # Hyper Priors
  beta0 ~ dnorm(0, tau.b0)
  beta1 ~ dnorm(0, tau.b1)
  beta2 ~ dnorm(0, tau.b2)
  beta3 ~ dnorm(0, tau.b3)
  beta4 ~ dnorm(0, tau.b4)
  beta5 ~ dnorm(0, tau.b5)
  beta6 ~ dnorm(0, tau.b6)
  beta7 ~ dnorm(0, tau.b7)

  # Individual random effects
  for(i in 1:M){
    ind.beta0[i] ~ dnorm(beta0, ind.tau.b0)
    ind.beta1[i] ~ dnorm(beta1, ind.tau.b1)
    ind.beta2[i] ~ dnorm(beta2, ind.tau.b2)
    ind.beta3[i] ~ dnorm(beta3, ind.tau.b3)
    ind.beta4[i] ~ dnorm(beta4, ind.tau.b4)
    ind.beta5[i] ~ dnorm(beta5, ind.tau.b5)
    ind.beta6[i] ~ dnorm(beta6, ind.tau.b6)
    ind.beta7[i] ~ dnorm(beta7, ind.tau.b7)}

  # hyperprior variance
  tau.b0 ~ dgamma(1,1)
  tau.b1 ~ dgamma(1,1)
  tau.b2 ~ dgamma(1,1)
  tau.b3 ~ dgamma(1,1)
  tau.b4 ~ dgamma(1,1)
  tau.b5 ~ dgamma(1,1)
  tau.b6 ~ dgamma(1,1)
  tau.b7 ~ dgamma(1,1)

  # individual level variance
  ind.tau.b0 ~ dgamma(1,1)
  ind.tau.b1 ~ dgamma(1,1)
  ind.tau.b2 ~ dgamma(1,1)
```

```
  ind.tau.b3 ~ dgamma(1,1)
  ind.tau.b4 ~ dgamma(1,1)
  ind.tau.b5 ~ dgamma(1,1)
  ind.tau.b6 ~ dgamma(1,1)
  ind.tau.b7 ~ dgamma(1,1)

  # likelihood model
  for(n in 1:nobs){
    y[n] ~ dbern(theta[n])
    logit(theta[n]) <- ind.beta0[id[n]] + # intercept
      ind.beta1[id[n]] * nss[n] +          # non-sage shrub cover
      ind.beta2[id[n]] * sage[n] +         # sagebrush cover
      ind.beta3[id[n]] * biomass[n] +      # herbaceous biomass
      ind.beta4[id[n]] * sdc[n] +          # standard deviation of terrain curvature
      ind.beta5[id[n]] * hli[n] +          # heat-load index
      ind.beta6[id[n]] * swe[n] +          # snow water equivalent
      ind.beta7[id[n]] * road[n]+          # distance to road (decay function)

  }})


# how many explanatory variables in beta
numVars = 8

# Initial values if desired
inits = list('set initial values as desired')

# Parameters to monitor
params = ('set parameters to monitor')

#Separate data and constants
Constants = list(M = max(df$id), id = df$id, nobs = dim(df)[1], numVars = numVars)

nim.data = list(y=df$used, nss = df$NSS, sage = df$SAG, biomass = df$biomass,
                sdc = df$sdcurv, hli = df$hli swe = df$swe, road = df$road500m_decay)

# Check list structure and dimensions of data
str(constants)
str(nim.data)

# Create the model object and configure for MCMC
glmmModel = nimbleModel(code = NimModel,
                        constants = constants,
                        data = nim.data,
                        inits = inits)
```

```
glmmModelc = configureMCMC(glmmModel,
                           monitors = params,
                           control = list(adaptInterval = 5000), thin=1)

# Create the default MCMC algorithm from the model object.
glmmMCMC = buildMCMC(glmmModelc)

## Compile the model and MCMC algorithm
CglmmModel = compileNimble(glmmModel)
CglmmMCMC = compileNimble(glmmMCMC, project = glmmModel)


## Execute MCMC algorithm and extract samples
samples = runMCMC(CglmmMCMC, niter = 35000,
          nburnin = 5000,
          nchains = 4,
          thin = 5,
          samplesAsCodaMCMC = TRUE)

# Review output
summary(samples)
```

Supplementary References

1. Jarvis, A., Guevara, E., Reuter, H. I. & Nelson, A. D. Hole-filled SRTM for the globe : version 4 : data grid. https://research.utwente.nl/en/publications/hole-filled-srtm-for-the-globe-version-4-data-grid/ (2008).
2. Janousek, W. M. *et al.* Predicted habitat suitability and associated covariate data used to explain changes in mule deer age ratios from 2001 to 2023, Wyoming, USA. U.S. Geological Survey https://doi.org/10.5066/P1SJAMX7 (2025).
