# Supplementary material for: The interplay of habitat quality and temperature shape demographic patterns of mule deer (Odocoileus hemionus) in North America
Source: Commun Biol. 2026 Apr 7;9:761. doi: 10.1038/s42003-026-09687-8 (PMC13233836; doi:10.1038/s42003-026-09687-8)
Supplement: Supplementary file 3 — Reporting Summary [file 42003_2026_9687_MOESM3_ESM.pdf]

nature portfolio

Corresponding author(s): William Janousek

Last updated by author(s): Jan 23, 2026

Reporting Summary

Nature Portfolio wishes to improve the reproducibility of the work that we publish. This form provides structure for consistency and transparency in reporting. For further information on Nature Portfolio policies, see our Editorial Policies and the Editorial Policy Checklist.

Statistics

For all statistical analyses, confirm that the following items are present in the figure legend, table legend, main text, or Methods section.

n/a	Confirmed	
☐	☒	The exact sample size (*n*) for each experimental group/condition, given as a discrete number and unit of measurement
☐	☒	A statement on whether measurements were taken from distinct samples or whether the same sample was measured repeatedly
☐	☒	The statistical test(s) used AND whether they are one- or two-sided<br>*Only common tests should be described solely by name; describe more complex techniques in the Methods section.*
☐	☒	A description of all covariates tested
☐	☒	A description of any assumptions or corrections, such as tests of normality and adjustment for multiple comparisons
☐	☒	A full description of the statistical parameters including central tendency (e.g. means) or other basic estimates (e.g. regression coefficient) AND variation (e.g. standard deviation) or associated estimates of uncertainty (e.g. confidence intervals)
☒	☐	For null hypothesis testing, the test statistic (e.g. *F*, *t*, *r*) with confidence intervals, effect sizes, degrees of freedom and *P* value noted<br>*Give P values as exact values whenever suitable.*
☐	☒	For Bayesian analysis, information on the choice of priors and Markov chain Monte Carlo settings
☐	☒	For hierarchical and complex designs, identification of the appropriate level for tests and full reporting of outcomes
☐	☒	Estimates of effect sizes (e.g. Cohen's *d*, Pearson's *r*), indicating how they were calculated

Our web collection on statistics for biologists contains articles on many of the points above.

Software and code

Policy information about availability of computer code

Data collection	Source datasets for resource selection and age ratio models and seasonal habitat suitability raster data are available in a data release cited within the main text. Raw mule deer GPS location data used in this study is part of a broad collaboration with many partners and interest in access to this information should be relayed to the corresponding author.
Data analysis	A description of the resource selection model formulated for use in NIMBLE is available in the Supplementary Information. No additional novel code was developed for these analyses; all software packages, versions, and programs used are documented within.

For manuscripts utilizing custom algorithms or software that are central to the research but not yet described in published literature, software must be made available to editors and reviewers. We strongly encourage code deposition in a community repository (e.g. GitHub). See the Nature Portfolio guidelines for submitting code & software for further information.

Data

Policy information about availability of data

All manuscripts must include a data availability statement. This statement should provide the following information, where applicable:

- Accession codes, unique identifiers, or web links for publicly available datasets
- A description of any restrictions on data availability
- For clinical datasets or third party data, please ensure that the statement adheres to our policy

Covariate and response variable datasets for resource selection and age ratio models and seasonal habitat suitability raster data are available in Janousek et al.

(2025; https://doi.org/10.5066/P1SJAMX7). Raw mule deer GPS location data used in this study is part of a broad collaboration with many partners and interest in access to this information should be relayed to the corresponding author.

Research involving human participants, their data, or biological material

Policy information about studies with human participants or human data. See also policy information about sex, gender (identity/presentation), and sexual orientation and race, ethnicity and racism.

Reporting on sex and gender	NA
Reporting on race, ethnicity, or other socially relevant groupings	NA
Population characteristics	NA
Recruitment	NA
Ethics oversight	NA

Note that full information on the approval of the study protocol must also be provided in the manuscript.

Field-specific reporting

Please select the one below that is the best fit for your research. If you are not sure, read the appropriate sections before making your selection.

☐ Life sciences ☐ Behavioural & social sciences ☒ Ecological, evolutionary & environmental sciences

For a reference copy of the document with all sections, see nature.com/documents/nr-reporting-summary-flat.pdf

Ecological, evolutionary & environmental sciences study design

All studies must disclose on these points even when the disclosure is negative.

Study description	We used GPS collar data from mule deer in Wyoming to fit seasonal resource selection functions, predict habitat suitability, and then modeled age ratios (fawn:doe) as a function of drought conditions, winter severity, and seasonal habitat suitability to gauge the relative influence of each of these factors in driving demographic rates in mule deer.
Research sample	The data we used was compiled in a large collaboration across many partner groups to pool previously collected data from prior projects in this region. In total, our data includes location information from 1473 female mule deer over 22 years resulting in 661486 unique randomly selected daily locations.
Sampling strategy	We structured our analysis binning collar data from individual deer into regional groups based on geographic distance and ecological similarity. These regional groupings were used to fit region-specific resource selection models because the predicted strength and direction of selection for resources may vary across space as the availability of those resources varies.
Data collection	No data collection was required. All data has been previously collected.
Timing and spatial scale	Data spans 22 years, 2001-2022, across the entire state of Wyoming, USA.
Data exclusions	Our analysis is focused on summer and winter periods so we excluded days of spring and fall migration for migratory deer. For non-migratory deer we used the median start/end migration dates of the migratory population to exclude dates of seasonal transition and delineate the summer and winter periods.
Reproducibility	This study uses a long-term observational dataset and was not setup as a reproducible experiment. However, we conducted a series of statistical tests to evaluate goodness-of-fit using k-fold cross validation.
Randomization	We selected one random used location per day per individual deer to reduce the dimensionality of the dataset and because GPS collars vary in their fix rates based on manufacturer and user settings.
Blinding	Blinding was not relevant to our study because it was not experimental.

Did the study involve field work? ☐ Yes ☒ No

Reporting for specific materials, systems and methods

We require information from authors about some types of materials, experimental systems and methods used in many studies. Here, indicate whether each material, system or method listed is relevant to your study. If you are not sure if a list item applies to your research, read the appropriate section before selecting a response.

Materials & experimental systems

n/a	Involved in the study
☒	☐ Antibodies
☒	☐ Eukaryotic cell lines
☒	☐ Palaeontology and archaeology
☐	☒ Animals and other organisms
☒	☐ Clinical data
☒	☐ Dual use research of concern
☒	☐ Plants

Methods

n/a	Involved in the study
☒	☐ ChIP-seq
☒	☐ Flow cytometry
☒	☐ MRI-based neuroimaging

Animals and other research organisms

Policy information about studies involving animals; ARRIVE guidelines recommended for reporting animal research, and Sex and Gender in Research

Laboratory animals	NA
Wild animals	All data in this study was previously collected following approved protocols and permitted procedures. This study is a collaboration across many partners to pool individual datasets which have been previously used in a range of scientific studies on mule deer over the last two decades.
Reporting on sex	NA
Field-collected samples	NA
Ethics oversight	NA

Note that full information on the approval of the study protocol must also be provided in the manuscript.

Plants

Seed stocks	NA
Novel plant genotypes	NA
Authentication	NA
